# Mechanism and application of injectable hydrogel as carrier system in the treatment of osteoarthritis

**DOI:** 10.3389/fbioe.2025.1636518

**Published:** 2025-10-01

**Authors:** Bing-Gang Zhang, Qiang Liu, Tao Ma, Jian-Jun Liu, Yan Zhang, Fang Liu, Xiao-Ming Wen, Duo-Xian Wang, Wei Jiang, Wen-Bo An

**Affiliations:** ^**1**^ Affiliated Hospital of Gansu University of Chinese Medicine, Lanzhou, China; ^**2**^ Ningxia Medical University, Ningxia, China; ^**3**^ Gansu Provincial Hospital of TCM, Lanzhou, China

**Keywords:** osteoarthritis, biomaterials, injectable hydrogels, drug delivery systems, disease therapy

## Abstract

Osteoarthritis (OA) is a disabling degenerative disease that affects synovial joints and leads to cartilage degeneration, which can cause progressive joint damage, chronic pain and functional loss. Because its specific pathogenesis is still unclear, conventional treatment methods are still difficult to achieve satisfactory therapeutic effects. Therefore, finding alternative new methods for treating OA remains a formidable challenge. Hydrogel is a kind of polymer material with good biocompatibility and biodegradability, and it is a new method for the treatment of osteoarthritis. Injectable hydrogel drug delivery platforms have shown many advantages in the treatment of OA, including improved biocompatibility, biodegradability, and low immunogenicity. Injectable hydrogels, as delivery systems, can deliver drugs to the joint cavity in a controlled manner and continuously release them, enhancing drug loading capacity and increasing sensitivity to improve therapeutic effects. This article summarizes the types of injectable hydrogels, analyzes their application as delivery systems in OA, and discusses the mechanisms of injectable hydrogels in the treatment of OA, such as anti-inflammation, anti-oxidative stress, and promotion of articular cartilage regeneration. Meanwhile, the deficiencies of injectable hydrogel drug delivery platforms in the OA field were summarized, and the future research directions in this field were discussed. Overall, injectable hydrogel drug delivery platforms show great potential in the treatment of OA. These innovative methods have brought new hope for the future treatment of OA and pointed out the direction for clinical application.

## 1 Introduction

**FIGURE 1:**
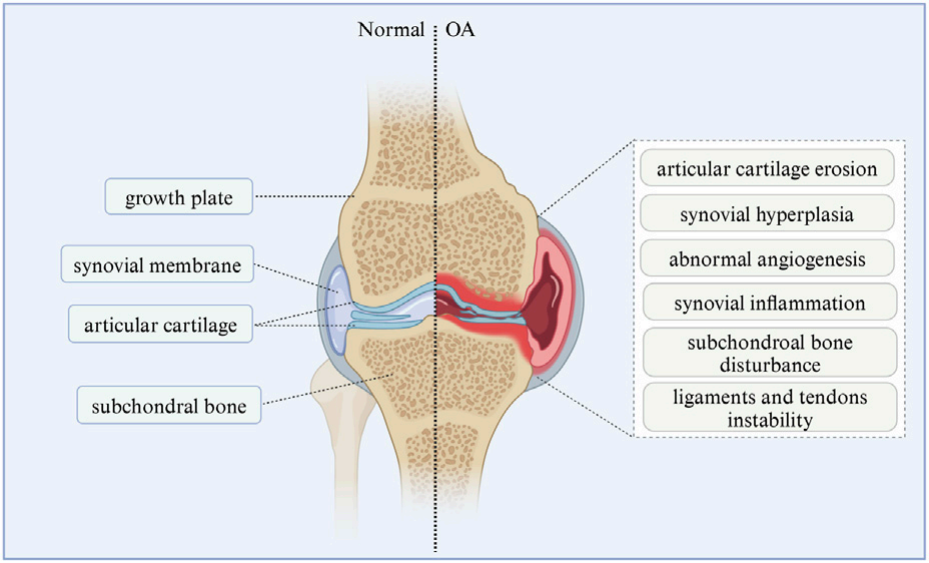
Pathological manifestations of normal joints and osteoarthritis. The left half shows the structure of the normal synovial joint, and the right half shows the structure and symptoms of synovial joint in osteoarthritis. The image is drawn using the BioRender software.

Osteoarthritis (OA) is one of the most common chronic degenerative and disabling diseases, characterized by complex disorders of the entire synovial joint, involving local cartilage loss, bone hyperplasia, synovial sac thickening, and structural changes of the periarticular ligaments and surrounding muscles (Hunter and Bierma-Zeinstra, 2019; Hunter et al., 2014) (Figure 1). It is estimated that about 500 million people worldwide suffer from OA, accounting for approximately 7% of the global population (Steinmetz et al., 2023). The pathogenesis of OA involves a variety of factors, including mechanical effects, effects of aging on the composition and structure of cartilage matrix, and genetic factors (Diamond et al., 2024; Dieppe and Lohmander, 2005). Its main clinical symptoms include chronic pain, joint instability, stiffness, and narrowing of joint space as shown by radiation (Chen D. et al., 2017). Without timely intervention and treatment, related symptoms and complications can lead to joint deformities and loss of function (Abramoff and Caldera, 2020). At present, the treatment of OA is mainly based on physical therapy and drug therapy based on rehabilitation exercise to reduce pain, reduce disease activity and prevent inflammation and destructive processes (Mattes and on, 2000; Arden et al., 2021). However, these methods can only relieve symptoms, but cannot reverse the course of the disease (Bowman et al., 2018).

Traditional drug therapies (nonsteroidal anti-inflammatory drugs (NSAIDs), opioids, and cyclooxygenase-2 (COX) specific drugs) only relieve symptoms without taking into account the underlying problem of cartilage disease. In addition, traditional therapies may cause side effects (especially with long-term use), which can reduce compliance and trigger multiple adverse reactions (Sengupta et al., 2008). To avoid these complications, intra-articular injection therapy became popular in the second half of the 20th century (Raynauld et al., 2003). For example, platelet-rich plasma (PRP) and mesenchymal stem cells (MSCs) are injected into the joint cavity to treat OA (Lana et al., 2023; An et al., 2024). Compared with traditional methods, intraarticular injection of synovial fluid has the advantages of high drug concentration and few side effects, but the effectiveness of intraarticular administration is limited due to poor drug permeability in cartilage, rapid clearance of components through synovial capillaries and lymphatics, and weakened synergies of active components in pathological microenvironments (Evans et al., 2014). For end-stage patients, joint replacement surgery is currently the only effective treatment method, but it also has some drawbacks, including high cost, risk of perioperative complications and postoperative periprosthetic infections, and the possibility of requiring revision of joint replacement surgery (Gunaratne et al., 2017). Due to the complex pathophysiological changes of OA and the harsh local microenvironment, a single treatment cannot repair the structure and function of the damaged joints. Therefore, there is an urgent need to explore innovative drug delivery systems to improve the therapeutic efficacy of OA. In recent years, injectable hydrogel systems have made remarkable progress in the field of biomedical applications (Oliveira et al., 2021). These biomaterials are injectable, biocompatible, biodegradable, and capable of matching irregular damage (Ding et al., 2023). Injectable hydrogels can be used as drug carriers directly or encapsulate smaller drug carriers to deliver drugs or biotherapeutic molecules accurately and in a controlled manner to the lesion site to provide safe and effective treatment and are widely used in the treatment of refractory diseases (Oliveira et al., 2021; Wang and Wang, 2021). Their potential has been demonstrated in various therapeutic areas, such as the treatment of joint diseases (Bruno et al., 2022), spinal cord injuries (Ji et al., 2023), degenerative diseases (Li P. et al., 2023), and tumors (Kim et al., 2022). In this article, we summarize the types and classifications of injectable hydrogels, as well as the potential applications of hydrogels as delivery systems in the treatment of OA, and emphasize the molecular mechanisms of hydrogel treatment for OA. Meanwhile, we also delved deeply into the limitations of current injectable hydrogels in the treatment of OA and proposed relevant solutions. The aim is to deeply explore the role and intrinsic mechanism of injectable hydrogels in the treatment of OA, providing new strategies and a theoretical basis for the clinical treatment of osteoarthritis.

## 2 Properties of injectable hydrogels for the treatment of OA

**FIGURE 2:**
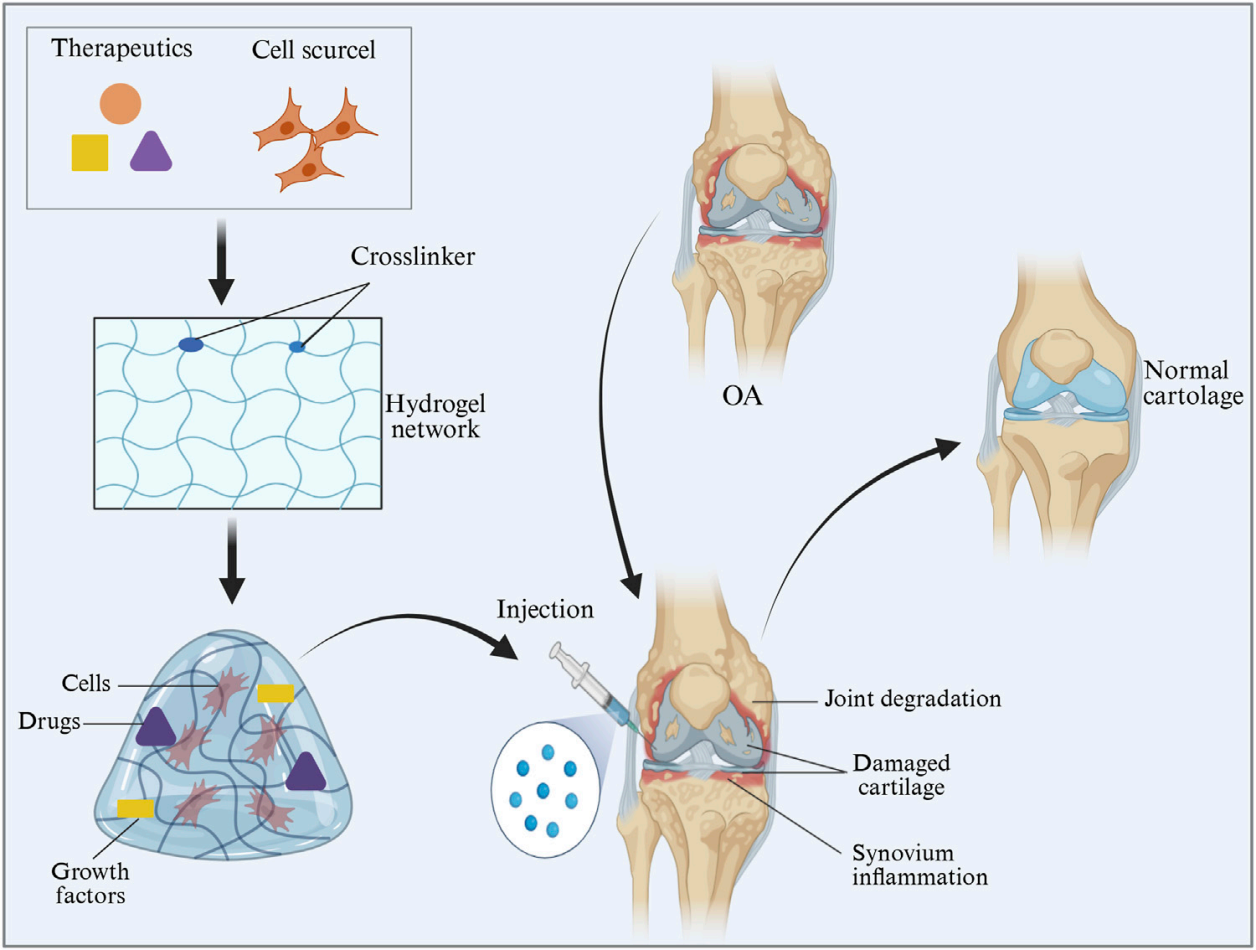
Feasibility diagram of injectable hydrogel in OA treatment. The image is drawn using the BioRender software.

In recent years, injectable hydrogel scaffolds have attracted wide attention in cartilage tissue engineering (Balakrishnan et al., 2011; Elisseeff, 2004). Hydrogels are three-dimensional polymer networks with significant expansibility and porosity, in which various solutes and nutrients can be located and able to diffuse (Chen et al., 2018; Peppas and Van Blarcom, 2016; Slaughter et al., 2009). Injectable hydrogels have unique biocompatibility and hydrophilicity, as well as the ability of phase transition-from sol to gel, forming a solid-like gel state (Jia et al., 2020; Chatterjee et al., 2018). It can be delivered non-invasively or minimally via direct injection or arthroscopy, helping to encapsulate and release drugs, genes, DNA, proteins, and cells in a continuously controlled manner (Nguyen et al., 2015). In addition, injectable hydrogels not only provide a biocompatible, biodegradable, and highly hydrated three-dimensional structure similar to the extracellular matrix of chondrocytes (ECM), but also improve the supply of nutrients and cellular metabolites through elastic properties (Frith et al., 2013; Jin et al., 2010). It can also encapsulate cells and effectively deliver bioactive molecules to target sites through a stimulus-response release mechanism (Li et al., 2012; Chen et al., 2016) (Figure 2). The ideal injectable hydrogel for OA treatment to promote cartilage regeneration and joint repair should generally meet the following criteria: (a) Easy to administer under physiological conditions, (b) guaranteed injectable (by chemical or physical cross-linking gelation during injection), (c) excellent biocompatibility and potential biodegradability, (d) able to mimic cartilage ECM characteristics and promote the chondrogenic potential of cells, (e) can easily fill the defect site of the joint and integrate with the surrounding natural cartilage tissue without displacement, and (f) has sustained release properties if related to local drug delivery (Yu and Ding, 2008; Rice et al., 2013; Mujeeb et al., 2013). Based on these characteristics, injectable hydrogels are promising candidates for the treatment of various diseases, including osteoarthritis.

## 3 Types of injectable hydrogels

Injectable hydrogels are usually formed by rapid sol-gel phase transition or *in situ* chemical polymerization and can be delivered directly to the target site by injection (Li et al., 2012). According to different standards, hydrogels can be classified considering the following parameters: According to the different sources of raw materials, hydrogels can be divided into natural polymer hydrogels and synthetic polymer hydrogels (Chao et al., 2020). Natural injectable hydrogels are usually composed of natural polymers such as polysaccharides, proteins, and DNA, and have excellent biocompatibility, biodegradability, and environmental sensitivity, but they are unstable and prone to degradation (Ishihara et al., 2019). Natural hydrogels typically outperform synthetic hydrogels in terms of long-term safety and immunogenicity, as their degradation products are easier to metabolize and have good biocompatibility, which can reduce the risk of immune responses (Pham et al., 2025; Pushpamalar et al., 2021). In addition, natural hydrogels exhibit lower immunogenicity in applications such as cartilage repair, allowing for better compatibility with human tissues (Wan et al., 2025; Mei et al., 2023). However, natural hydrogels have weaker mechanical elasticity and usually require compounding or cross-linking to enhance their mechanical properties (Shukla et al., 2025). In contrast, synthetic polymer hydrogels are composed of polymers with good biocompatibility and biodegradability, such as peptides and polyesters, synthesized through ring-opening polymerization reactions (Chao et al., 2020). Synthetic hydrogels have advantages in mechanical strength and elasticity, maintaining stability under high intensity and long-term load environments (Ji D. et al., 2024; Li D. et al., 2020). However, compared to natural hydrogels, synthetic hydrogels have poorer biocompatibility, biological activity, and biodegradability, and their degradation products may cause adverse reactions in body tissues, activating the immune system (Stevens and George, 2005). Therefore, natural hydrogels are more suitable for applications requiring biocompatibility and low immunogenicity, while synthetic hydrogels are more suitable for situations requiring higher mechanical strength and customized properties.

**FIGURE 3:**
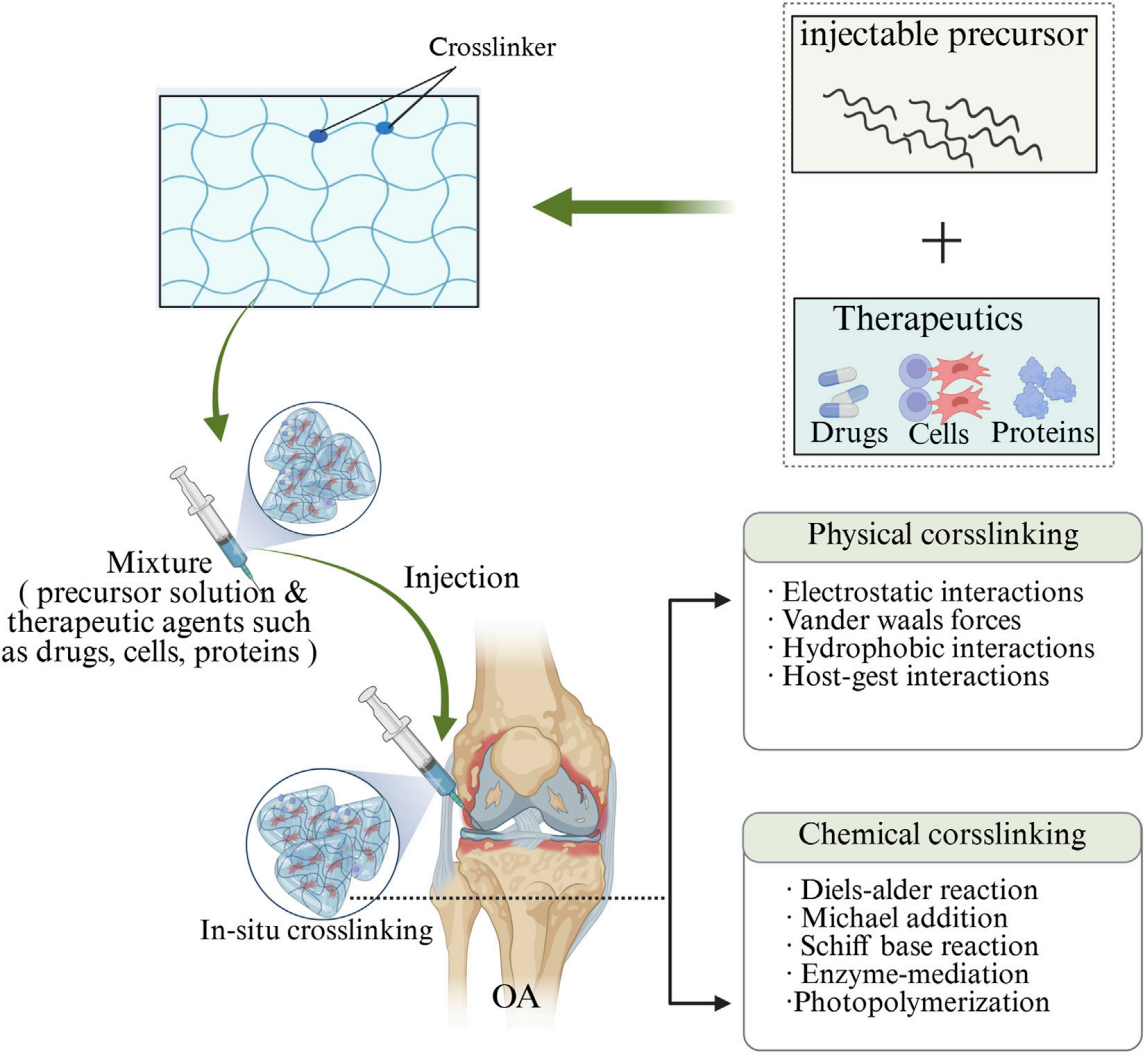
Diagram depicts the crosslinking of injectable hydrogels with therapeutic agents, such as small molecules, proteins and cells. Injectable hydrogels can be crosslinked using two common methods: physical crosslinking and chemical crosslinking. Physical crosslinking involves the formation of non-covalent bonds, such as electrostatic interactions, hydrogen bonds, and host-guest interactions. Chemical crosslinking, on the other hand, utilizes covalent bonds through a variety of strategies, including click chemistry, Schiff base reactions, enzyme-mediated reactions, and photopolymerization. The image is drawn using the BioRender software.

Additionally, according to the response of injectable hydrogels to external stimuli, injectable hydrogels can be divided into common hydrogels and smart hydrogels. Common injectable hydrogels are insensitive to environmental changes, while smart injectable hydrogels are affected by temperature, pH, enzymes, and photonics (Fan et al., 2019). Furthermore, based on the mechanism of forming three-dimensional network structures, injectable hydrogels can be classified into chemically cross-linked hydrogels and physically cross-linked hydrogels (Overstreet et al., 2012; Yang et al., 2014) (Figure 3). Chemically crosslinked hydrogels achieve *in situ* covalent cross-linking by chemical cross-linking reactions, most often through the chemical bond cross-linking between polymer chains exchanged by Michael addition reaction, photopolymerization, enzymatic reaction or mercaptan disulphide bond, thus forming matrix macromolecular structures (Zarembinski et al., 2014; Muzzarelli, 2009). Chemically cross-linked hydrogels have stable covalent cross-linked networks, so they have high mechanical strength and physical stability, long degradation time, and adjustable structure (Nada et al., 2019). While physical crosslinked hydrogels usually use non-covalent interactions (such as hydrophobic interactions, hydrogen bonding and ionic crosslinking) to cause polymer conformation changes and phase separation, resulting in polymer chain aggregation to form physical crosslinked networks (Wang et al., 2015; Ulijn et al., 2007; Yu et al., 2018). Since non-covalent bonds between molecules are easily broken, physically crosslinked hydrogels usually exhibit reversible sol-gel transition behavior (Moon et al., 2012). In addition, physically cross-linked hydrogels usually provide a friendly environment for cells and bioactive molecules, and they also exhibit relatively low mechanical strength, dynamic reversibility, no need for crosslinking agents, and repeatability, compared with physically cross-linked hydrogels (Liu Y. et al., 2018; Norouzi et al., 2016).

However, different types of hydrogels have their own advantages and challenges in clinical applications. Natural polymer hydrogels have good biocompatibility and excellent degradability, but their mechanical strength is relatively low and they are prone to degradation; synthetic polymer hydrogels possess higher mechanical strength and stability, but have poorer biocompatibility and degradability. Chemically cross-linked hydrogels provide strong stability and high mechanical strength, making them suitable for long-term use, but their degradation rate is slow, which may affect drug release; on the other hand, physically cross-linked hydrogels have better reversibility and biocompatibility, but their stability is poor and mechanical strength is low (Zhao et al., 2022; Khan et al., 2022; Segneanu et al., 2025). Smart hydrogels can respond to environmental changes and have flexible control capabilities, making them suitable for personalized treatment, but they are difficult to produce and have higher costs; ordinary hydrogels offer better stability but lack dynamic response, resulting in a more limited range of applications (Liu et al., 2019). These factors may lead to potential reasons for the failure of hydrogels in clinical use. Overall, the selection of hydrogels should be based on specific clinical needs, balancing biocompatibility, stability, mechanical strength, and responsiveness, to ensure therapeutic effectiveness (Li et al., 2025; Giuri et al., 2019).

## 4 Composition of injectable hydrogels for OA treatment

Hydrogels can be described as cross-linked polymer networks that restrict the flow of water inside. Therefore, the physical and chemical properties of polymers directly affect the properties of hydrogels. Both natural polymers (such as proteins and polysaccharides) and synthetic polymers (including polyvinyl alcohol (PVA), polyethylene glycol (PEG), and poly-isopropylacrylamide) have been used to construct injectable hydrogels for the treatment of joint diseases.

### 4.1 Natural polymer hydrogels

Natural polymers have been shown to be beneficial for tissue engineering applications, as they retain their biochemical properties and improve their biocompatibility with host tissues (Jawad et al., 2008). Common natural polymers used in joint therapy include hyaluronic acid, alginate, and chitosan (Li et al., 2013; Yoon et al., 2009; Rocca et al., 2016). These materials are composed of proteins and/or polysaccharides that can absorb water and expand, allowing nutrients and waste to easily diffuse through the natural polymer hydrogel scaffold, thereby enhancing cell survival rate and cell migration to surrounding tissues (Ahmed, 2015; Ahearne, 2014).

#### 4.1.1 Collagen

Collagen is the main component of cartilage tissue and the core structural protein of cartilage ECM. Its triple helical structure (the Gly-X-Y repeat sequence) forms a fibrous network hydrogel through hydrogen bonds and hydrophobic interactions (Wang et al., 2023). This hydrogel self-assembles into a three-dimensional network structure. The injection of this hydrogel into the joint space can maintain joint lubrication and reduce friction in the knee joint to the greatest extent (Qiao et al., 2021). In addition, this hydrogel is biocompatible and biodegradable, promoting the adhesion, growth, and differentiation of chondrocytes, thereby contributing to articular cartilage repair (Zhang et al., 2022). With recent technological advancements, improvement strategies for collagen hydrogels are continuously emerging. For instance, genetic engineering techniques that insert cartilage-inducing peptides such as KELPASVSS have provided new ideas for the functionalization of collagen hydrogels. This technology can enhance the cellular adhesion and differentiation-inducing abilities of hydrogels, thereby improving repair efficacy (Majumder et al., 2024). Besides genetic engineering approaches, the combined application of collagen hydrogels and other biomaterials also shows broad prospects, such as combining with hyaluronic acid to enhance biocompatibility and lubricating performance. These technological improvement strategies offer new possibilities and directions for the application of collagen hydrogels in cartilage repair (Tang et al., 2025; An et al., 2025).

#### 4.1.2 Hyaluronic acid

Hyaluronic acid (HA) is a linear glycosaminoglycan composed of β-1, 3-n-acetylglucosamine and β-1, 4-glucuronic acid disaccharide units in the extracellular matrix (Xu et al., 2012). The negative charge and hydroxyl group give hyaluronic acid molecules hydrophilic properties. When the temperature rises, the hydrophilic and hydrophobic components on the chain of the grafts of highly hydrophobic polymers interact, and the viscosity increases to form hydrogels (Tan et al., 2009). This hydrogel can absorb and retain a lot of water, forming a viscous environment similar to joint synovial fluid, providing good lubrication and cushioning for joint cartilage, and effectively reducing friction damage during joint movement. In addition, hyaluronic acid has the ability to reduce cellular inflammatory response and heal diseased tissues, as well as good biocompatibility, biodegradability, and excellent gel formation properties, and can affect cell behavior during tissue regeneration, making it promising in biomedical-related hydrogel systems (Sepulveda et al., 2023). However, HA hydrogels still have deficiencies in degradation rate and mechanical properties, which limits their effectiveness in long-term use. Therefore, researchers are exploring optimization strategies for drug sustained-release systems to enhance the clinical application effects of HA hydrogels. For instance, combining bioactive molecules (such as growth factors) with HA hydrogels can achieve more lasting therapeutic effects (Mao et al., 2025; Zhu et al., 2023).

#### 4.1.3 Chitosan

Chitosan is a naturally occurring polysaccharide composed of glucosamine and N-acetylglucosamine and is an excellent gelling agent. Chitosan has excellent biocompatibility, biodegradability, and antibacterial activity and can effectively resist bacterial infections in the synovial cavity, thereby reducing the risk of inflammation (Sugiyan et al., 2018; El-Saadony et al., 2025). The amino groups in its molecular structure confer a positive charge in an acidic environment and enable interaction with negatively charged biomolecules, such as nucleic acids and proteins, providing a potential platform for the combined application of gene therapy and cell therapy. Chitosan degradation products, including Low-molecular-weight oligosaccharides and amino sugars, etc, can be metabolized; moreover, these products are readily formed into hydrogels (Martins et al., 2014; Delmar and Bianco-Peled, 2016). The preparation methods of chitosan hydrogels are diverse, including chemical cross-linking, physical cross-linking, and enzymatic cross-linking, among others (Cheng et al., 2023). Furthermore, the properties of pH-responsive gels are crucial. The pH-responsiveness of chitosan hydrogels makes them particularly advantageous for cartilage repair. These gels can exhibit different physical properties in various physiological environments, such as rapidly dissolving under acidic conditions or forming stable network structures under neutral or alkaline conditions (Zahedi Tehrani et al., 2024). This environmental responsiveness allows the hydrogels to better adapt to changes within the organism and enhance cartilage repair efficacy.

#### 4.1.4 Alginate

Sodium alginate is a natural polysaccharide derived from brown algae whose unique ionic cross-linking properties enable it to undergo a rapid gelation transition in the presence of divalent cations such as Ca^2+^, forming a stable three-dimensional network structure. This gelation process is gentle and controllable, providing convenient conditions for drug loading and cell encapsulation (encapsulating living cells within biocompatible materials to form a physical isolation barrier to protect the cells and promote their growth and function in a specific environment). Meanwhile, this structure can better simulate the structural characteristics of biological tissues and promote the growth and differentiation of chondrocytes (Ma et al., 2019; Chu et al., 2021). The application of saline alginate gel in cartilage tissue engineering has received increasing attention, especially in the construction of three-dimensional culture environments.

In the treatment of OA, anti-inflammatory drugs or growth factors can be loaded onto sodium alginate hydrogels. By taking advantage of their slow release characteristics, continuous inhibition of local joint inflammation and directional induction and differentiation of chondrocytes can be achieved, promoting cartilage repair (Yu et al., 2023). However, constructing an efficient drug delivery system is another important application of alginate gel in cartilage repair. The sustained-release kinetics of drugs is influenced by multiple factors, including the physicochemical properties of the drugs, the cross-linking degree of the gel, and environmental conditions, etc. (Zhang W. et al., 2025). To achieve controlled release of drugs, modified alginate gel is often used. This modification can enhance the loading capacity and release control ability of drugs (Liang et al., 2025). However, in the process of controlled release of drugs, how to balance the release rate and biological effects remains a technical difficulty. Current research is still constantly exploring new technologies to enhance the efficiency of drug delivery systems, such as the combined application of nanocarriers or bioactive factors. These new technologies are expected to improve the bioavailability and therapeutic effects of drugs (Yin P. et al., 2023).

### 4.2 Synthetic polymer hydrogels

The development of synthetic polymers aims to eliminate the undesirable properties of natural polymers while retaining their ideal characteristics (Ozdil et al., 2014; Place et al., 2009). The desired synthetic polymer hydrogels can be obtained by altering mechanical strength, porosity, degradation rate, gelation rate, and other polymer properties. This synthetic polymer hydrogel features excellent flexibility, durability, and biocompatibility, which makes controlling its properties easier and reduces the risk of immune rejection post-implantation (Patel et al., 2024a).

#### 4.2.1 Polyethylene glycol

Polyethylene glycol (PEG) is a biocompatible synthetic polymer widely used in tissue engineering methods (Zhu, 2010). PEG can be copolymerized with biocompatible polyesters to prepare thermosensitive hydrogels. The thermosensitivity of hydrogels can be improved by adjusting the composition and length of hydrophilic PEG blocks and hydrophobic polyester blocks. PEG is mixed with polylactic acid and poly (lactide-co-glycolide) (PLGA) to form the b-a-b triblock copolymer PLGA-PEG-PLGA. The temperature sensitivity of copolymers is controlled by the hydrophilic and hydrophobic groups in the polymers (Chang et al., 2007). At low temperatures, hydrophobic cores and hydrophilic shells form micelles through self-assembly. As the ambient temperature rises, the shell dehydrates, micelle aggregation increases, and the copolymer undergoes sol-gel and gel-sol transitions (Yu et al., 2009). Relevant studies also show that hydrogels synthesized using PEG and PLGA perform well in terms of drug loading, release rate, and biocompatibility, thus having broad application potential in fields such as anti-cancer treatment, local anesthesia, and regenerative medicine (Lei et al., 2021; Kumar et al., 2022). In addition, an important advantage of PEG hydrogels is that they can be administered painlessly by injecting low-viscosity precursor solutions, providing patients with a more comfortable medication experience (Bakaic et al., 2015). Studies have shown that the low viscosity property of PEG hydrogels enables them to flow rapidly after injection and fill the target site, achieving precise drug release. For instance, injectable PEG hydrogels can be used to treat bone defects due to their excellent biocompatibility and biodegradability, effectively promoting bone regeneration without causing significant discomfort to patients during administration (Sun et al., 2023). Therefore, by adjusting the physicochemical properties of PEG hydrogels, more precise and effective drug delivery can be achieved. This can improve therapeutic effects, reduce patient discomfort, and offer new strategies for personalized and precise drug treatment.

#### 4.2.2 Polyvinyl alcohol (PVA)

Polyvinyl alcohol (PVA) is an excellent water-soluble synthetic polymer and is widely used in the biomedical field. PVA hydrogels with stable three-dimensional network structures can be formed through physical crosslinking methods or chemical crosslinking (Zhong et al., 2024). This structure not only endows PVA with excellent mechanical properties, but also makes it perform outstandingly in terms of water absorption and biocompatibility. Research has found that the crosslinking density and hydration state of PVA hydrogels directly affect their mechanical properties and biological behaviors. Appropriate crosslinking can significantly enhance the strength and durability of hydrogels, making them more advantageous in biomedical applications (Liu et al., 2023; Madfoon et al., 2024). The low-friction behavior of PVA hydrogel is similar to that of cartilage tissue. This feature makes it an ideal substitute for articular cartilage, effectively buffering and shock-absorbing, and alleviating joint inflammation and pain (Chen et al., 2021). However, PVA hydrogels exhibit relatively low biological activity, and their mechanical strength and durability may not be sufficient to withstand long-term joint pressure and wear, thereby limiting their application (Zhao et al., 2020). To address this issue, researchers began to explore ways to enhance the mechanical properties of PVA hydrogels by adding reinforcing materials such as biodegradable glass fibers. Research has found that the compressive strength of PVA composite hydrogels doped with biodegradable glass fibers can reach 3.05 MPa, approaching the mechanical properties of human articular cartilage. This provides new possibilities for the clinical application of PVA hydrogels in cartilage repair (Zhu C. et al., 2022).

#### 4.2.3 Poly (N-isopropylacrylamide)

Poly (N-isopropylacrylamide) (PNIPAm) is a widely studied thermoresponsive polymer and one of the most thoroughly investigated thermosensitive polymers in biomedical applications (Karimi et al., 2016). The chemical structure of PNIPAm consists of hydrophilic amide groups and hydrophobic isopropyl side chains. This amphiphilic structure allows PNIPAm’s properties to be finely tuned. Studies have shown that the LCST of PNIPAm is approximately 32 °C, which enables reversible thermoresponsive phase transition near body temperature. Specifically, it is a transparent aqueous solution below the LCST but rapidly transforms into a hydrophobic collapsed state when the temperature rises above the LCST (Narayana et al., 2025). This property enables thermoresponsive drug release, which can potentially enhance the bioavailability of drugs and reduce side effects (Ansari et al., 2022). Consequently, the thermoresponsive phase transition characteristics of PNIPAm are also utilized in the design of cell culture scaffolds, allowing cells to attach or detach rapidly when the temperature changes. This characteristic is particularly suitable for applications requiring non-invasive cell collection, such as stem cell culture and regenerative medicine (Yang et al., 2024; Zhang Z. et al., 2025). Beyond its use in drug carriers and cell culture scaffolds, PNIPAm is also widely applied in tissue engineering. Its adjustable mechanical properties and excellent biocompatibility make it an ideal scaffold material capable of supporting cell growth and differentiation (Kotova et al., 2023). For instance, research indicates that combining PNIPAm with other natural polymers can enhance its mechanical strength and biodegradability, thereby improving its functional performance in tissue engineering (Raghuwanshi et al., 2023).

#### 4.2.4 Poloxam

Poloxamer is a type of water-soluble nonionic triblock copolymer, mainly composed of polyethylene oxide (PEO) and polypropylene oxide (PPO). This unique structure makes Poloxamer’s behavior in aqueous solutions temperature-sensitive (Fu et al., 2015). In such solutions, these copolymers form ordered micelles at appropriate temperatures and concentrations (Bodratti and Alexandridis, 2018; Thapa et al., 2020). As the temperature rises, micellar aggregation increases, leading to a sol-gel transition. At low temperatures, Poloxamer usually exists in liquid form with good fluidity, facilitating its administration via injection. Once the temperature reaches or exceeds its critical gel temperature (CGT)—for instance, at body temperature (37 °C)—Poloxamer rapidly transforms into a gel state. This process not only provides sustained local drug release but also enables continuous drug action at the target site, reducing drug clearance caused by fluid flow (Uk Son et al., 2020). Additionally, the high water content and favorable rheological properties of Poloxamer allow it to form injectable hydrogels, greatly reducing surgical risks in clinical applications (Chen et al., 2013). Studies have shown that thermosensitive hydrogels based on Poloxamer turn into a viscous gel state at body temperature. This effectively maintains the local drug concentration at the injection site and provides prolonged drug release rather than rapid diffusion to other areas. These characteristics make Poloxamer highly promising for application in local drug delivery systems, especially for diseases requiring precise targeted treatment, such as tumors and chronic pain (Abdeltawab et al., 2020). Moreover, Poloxamer demonstrates excellent performance in regulating the drug release rate. By adjusting its concentration and temperature conditions, the drug release rate can be effectively controlled, providing the possibility for personalized treatment (Rafiee et al., 2024).

In all types of hydrogels, collagen hydrogels, hyaluronic acid hydrogels, chitosan hydrogels, alginate hydrogels, PEG hydrogels, and Poloxamer hydrogels are all highly promising types. They provide effective treatment options in the fields of OA and cartilage repair due to their good biocompatibility, biodegradability, lubricity, and ability to control drug release (Niu et al., 2009). With the continuous advancement of technology, especially driven by functional design and combination therapy, the prospects of these hydrogels in clinical applications will be even broader (Wang F. et al., 2021; Wu et al., 2020). However, hybrid hydrogels are most likely to become the main choice for next-generation OA treatments in future research, as they combine the advantages of natural and synthetic hydrogels (Mohanty et al., 2024; Gupta et al., 2022). By providing excellent biocompatibility and biodegradability through natural polymers, while also incorporating the mechanical strength and controllability of synthetic polymers, hybrid hydrogels can offer ideal mechanical properties and stability. Their adjustable degradation rate and drug delivery characteristics allow for precise control of drug release according to treatment needs and timely degradation after repair (Patel et al., 2024b). In addition, the flexibility of hybrid hydrogels also supports personalized treatment, meeting the needs of different patients. Therefore, it shows broad application potential in cartilage repair, joint protection, and drug delivery (Xu et al., 2025; Arif et al., 2025).

## 5 Application of hydrogels as delivery systems in the treatment of OS

**FIGURE 4:**
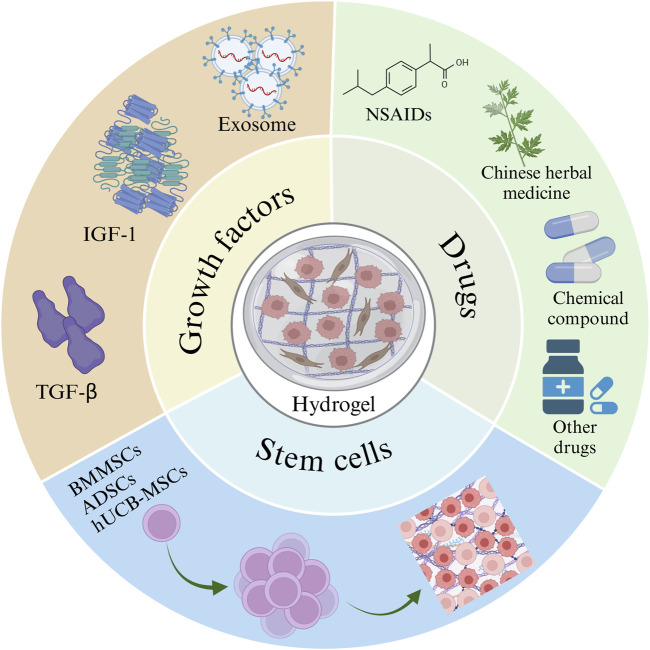
Hydrogels rich in stem cells, drugs, and growth factors. The image is drawn using the BioRender software.

Injectable hydrogels are prepared by mixing a drug with a temperature-responsive polymer to form a flowable solution or suspension. After injecting this hydrogel precursor solution into the lesion site, a sol-gel transition can occur at body temperature to form a gel. The resulting hydrogel remains at the disease site, enabling sustained drug delivery. Due to their *in situ* encapsulation and minimally invasive delivery capabilities, injectable hydrogels can serve as drug delivery carriers that encapsulate and deliver various substances, such as cells, drugs, and biomolecules (Oliva et al., 2017; Li et al., 2021; Miller et al., 2021) (Figure 4).

### 5.1 Drugs

Although many drugs have been tested in experimental models of OA, the current primary mode of administration is by mouth (Zhang et al., 2016). These traditional methods have low bioavailability and poor absorption, and drugs cannot have a direct effect on the lesion site (Qindeel et al., 2020). Based on the fact that oral drugs do not work well and can cause harmful side effects, injectable hydrogel treatments for drug delivery are proposed. Injectable hydrogels can overcome and optimize the shortcomings of these traditional methods, and injectable hydrogels can be used as carriers to introduce drugs or natural active substances into the lesion site (Almawash et al., 2022; Walsh et al., 2022). This makes injectable hydrogels an excellent candidate for developing an intra-articular controlled release platform.

#### 5.1.1 Glucocorticoid

Glucocorticoids are commonly used drugs in osteoarthritis (OA) treatment; they can relieve pain quickly and effectively and have been widely used in clinical practice. Dexamethasone is an important glucocorticoid in OA treatment, and it can relieve joint symptoms and has a cartilage-protective effect (Huebner et al., 2014). Although intra-articular (IA) injection can enhance bioavailability and reduce off-target effects, rapid clearance of therapeutic drugs remains a problem (Zhao et al., 2019). Therefore, researchers have focused on developing preparations that prolong the retention of active molecules in joints.

The injectable thermosensitive hydrogel was prepared by physically blending chitosan and Pluronic F127. This thermosensitive property makes the hydrogel fluid at room temperature for easy injection. At body temperature, it turns solid, thereby achieving local drug release. This hydrogel has good biocompatibility and low cytotoxicity, supporting its potential application in the treatment of osteoarthritis and related diseases (Kankariya and Chatterjee, 2023; Chakravarti and Joseph, 2025). García-Couce J et al. used the hydrogel as a delivery carrier for dexamethasone and injected it into the knee joints of collagenase-induced OA mice. They found that the hydrogel could prolong the retention time of dexamethasone in the joint space and reduce its diffusion into surrounding normal tissues and organs, thus effectively delaying bone destruction in the joint and reducing inflammation in mice (García-Couce et al., 2022).

Chitosan-borax-glycerol hydrogel is a drug delivery system with temperature-induced phase transition properties. This hydrogel can change from liquid to solid at body temperature, thus forming a stable carrier in the joint space and enabling sustained local drug release. This phase transition property allows the hydrogel to solidify rapidly after injection, forming a protective barrier and prolonging the retention time of the drug in the joint, which enhances therapeutic efficacy (Kalairaj et al., 2024). Wang et al. loaded this hydrogel with dexamethasone and injected it into the knee joints of OA rats. The hydrogel effectively prolonged the retention time of dexamethasone in the joint space, reduced its diffusion into the blood, minimized potential side effects associated with low-frequency treatment, and achieved high and sustained local drug concentrations. This treatment effectively reduced bone destruction in the joints and slowed the progression of OA (Wang Q. S. et al., 2021; Wu et al., 2017).

#### 5.1.2 Non-steroidal anti-inflammatory drugs (NSAIDs)

NSAIDs are typically described as inhibitors of cyclooxygenase (COX), which is involved in the metabolism of arachidonic acid and the generation of prostaglandins. During treatment, NSAIDs often cause side effects such as gastrointestinal reactions and kidney damage (Davies et al., 2000). To avoid these adverse reactions, scholars used alginate and poloxamer to form three-dimensional network structure hydrogels through ionic cross-linking reactions. This hydrogel formed a safe and effective drug delivery platform, demonstrating excellent sustained-release properties (Hu et al., 2021). Alginate-poloxamer hydrogel has shown a good cartilage protective effect in the treatment of osteoarthritis. Studies have shown that hydrogels can effectively inhibit the apoptosis of chondrocytes and promote the synthesis of collagen, thereby enhancing the regenerative capacity of cartilage (Ferreira et al., 2023). Moreover, *in vitro* and *in vivo* experiments have found that the sustained release of indomethacin can enhance the survival rate of chondrocytes and significantly increase collagen synthesis. In addition, when this hydrogel is applied *in vivo*, it can effectively reduce inflammatory factors in the joint cavity, thereby lowering local inflammatory responses and promoting the repair and regeneration of cartilage (Díaz-Rodríguez and Landin, 2015; Dang et al., 2021).

Sodium diclofenac, as a non-steroidal anti-inflammatory drug, can effectively inhibit the synthesis of prostaglandins, thereby reducing inflammation and pain. Studies have shown that when chitosan hydrogel is used to deliver diclofenac sodium, sustained drug release can be achieved, which helps maintain the effective concentration of the drug in the body and thereby enhances its anti-inflammatory and analgesic effects (Wu et al., 2025). For instance, by using injectable chitosan hydrogels, the release of diclofenac sodium can continue for several hours or even days. This sustained-release property can effectively reduce the frequency of medication for patients and lower the side effects of the drug. Furthermore, experimental results show that the hydrogel delivery system can significantly enhance the local bioavailability of the drug, thereby achieving a higher drug concentration at the inflammatory site and further enhancing its therapeutic effect (Hoang et al., 2022). These findings provide important theoretical and experimental support for the application of chitosan hydrogel-based drug delivery systems in chronic conditions such as osteoarthritis.

#### 5.1.3 Native compound

In recent years, natural compounds such as icariin (ICA) and curcumin have received widespread attention as alternative and effective means to treat OA. ICA is the main bioactive component of the Chinese herb *Epimedium* and has extensive pharmacological effects (Li et al., 2015; Feng et al., 2019). ICA can significantly induce stem cells to differentiate into chondrocytes, promote chondrocyte proliferation and the related gene expression, reduce the expression of matrix metalloproteinases (MMPs), and enhance the secretion of the ECM, thus enhancing cartilage repair (Wang et al., 2018). A biodegradable hydrogel loaded with ICA was prepared through *in situ* cross-linking of the hyaluronate-calcium complex (HA-Ca) and sodium alginate (Alg-Na). This hydrogel can promote chondrocyte proliferation, inhibit cartilage matrix degradation, alleviate inflammation and pain, and protect chondrocytes, thereby delaying osteoarthritis (Zheng et al., 2025). Zhu et al. prepared hydrogels containing different concentrations of ICA by *in situ* cross-linking of hyaluronic acid and Poloxamer 407. Intra-articular injection of this hydrogel can promote the proliferation of BMSCs and their differentiation into chondrocytes through the Wnt/β-catenin signaling pathway. This process effectively repairs damaged cartilage tissue and slows down the progression of OA (Park et al., 2022). In addition, this hydrogel can effectively inhibit the expression of inflammatory factors in the OA model, thereby significantly alleviating the inflammatory response of the joint and relieving pain (Chen et al., 2020).

Curcumin is a natural polyphenolic compound extracted from turmeric, possesses various pharmacological activities (AloK et al., 2015). Curcumin has anti-inflammatory effects, and has recently been widely studied for the treatment of OA (Zheng et al., 2015). However, due to the low solubility of curcumin in aqueous solution, its systemic bioavailability is poor, which greatly hinders its therapeutic effect and clinical translation (Gunaratne et al., 2017). To enhance the water solubility of curcumin, researchers have employed a variety of technical approaches, among which the most effective is loading curcumin into a hydrogel composed of polyethylene glycol (PEG) and gelatin methacrylate (GelMA). The preparation of PEG-GelMA hydrogel usually involves a photoinitiated polymerization reaction. This process can not only effectively encapsulate curcumin in the hydrogel network but also maintain its biological activity (Lopresti, 2018). In the rabbit OA model, after loading curcumin into PEG-GelMA hydrogel, the damaged cartilage area showed good signs of healing. Indicators of chondrocyte proliferation and differentiation, such as cell viability and expression of cartilage-specific markers, were significantly improved. The thickness and surface smoothness of the cartilage were significantly enhanced, and higher levels of cartilage matrix synthesis were observed in histological analysis compared to controls. These results indicate that curcumin not only effectively alleviates the inflammation caused by arthritis but also improves joint function by promoting cartilage regeneration (Sun et al., 2022). At present, research on injectable hydrogels loaded with natural compounds for the treatment of OA is relatively limited. However, through in-depth studies of the mechanisms underlying cartilage repair and anti-inflammatory effects, a foundation can be laid for future clinical applications, providing patients with better treatment options.

### 5.2 Stem cell

Cell therapy is the transplantation of living cells into defective tissues or organs *in vivo* to restore their original function. Stem cell transplantation is a common form of cell therapy (Yamanaka, 2020). Stem cells are cells with self-renewal and multipotent differentiation potential, which can repair damaged tissues, improve the microenvironment, and promote tissue regeneration (Zakrzewski et al., 2019; Giorgino et al., 2023). Stem cell therapy was developed alongside drug-loaded injectable hydrogel therapy and plays a crucial role in joint diseases (Lopez-Santalla et al., 2021; Sabi et al., 2022; Burdick et al., 2016). Hydrogels protect transplanted stem cells from host inflammation by providing physical support that helps maintain their position in the injured area (Huang et al., 2017). Meanwhile, stem cells stimulate damaged tissues to form a balanced inflammatory and regenerative microenvironment by secreting therapeutic regenerative bioactive factors (Shi et al., 2018; Collins et al., 2023). Therefore, injectable hydrogel-loaded stem cells may become a promising method in the treatment of OA.

#### 5.2.1 Bone marrow mesenchymal stem cells (BMMSCs)

Mesenchymal stem cells (MSCs) therapies have shown good promise in regenerative medicine and have been successfully used in preclinical models. In early clinical trials, MSCs administered via intra-articular injection can migrate chemotactically to the injured area to secrete growth factors and extracellular matrix molecules, promoting cartilage regeneration and cell proliferation (Gonzalez-Fernandez et al., 2024; Mancuso et al., 2019). However, due to the microenvironment of the lesion site—such as inflammation, oxidative stress, and mechanical forces—stem cells cannot attach to the damaged joint to form functional networks. This prevents them from remaining and surviving in the lesion site for an extended period, thereby limiting the expected therapeutic effect (Hashimoto et al., 2023). Hydrogels are ideal biomaterials for assisting the delivery of MSCs. The combination of hydrogels and cell delivery systems can stabilize the cells at the injured site and provide the attachment sites necessary for stem cell survival, directly addressing the challenges of viability and retention of transplanted cells. Consequently, this approach improves cell viability after delivery and prolongs the retention time of stem cells in the target area (Suzuki et al., 2023; Baldari et al., 2017; Soto-Gutierrez et al., 2010).

Bone marrow-derived mesenchymal stem cells (BMMSCs) can improve pain relief and repair knee function, and are considered a promising therapeutic alternative (Orozco et al., 2013). Currently, the combination of BMMSCs with various structures prepared from natural or synthetic materials has been extensively studied in the medical field (Liao et al., 2014; Seo et al., 2014). For example, Liu et al. found that PEG-polypeptide triblock copolymer hydrogels enhanced the adhesion and proliferation of BMMSCs *in vitro*, and mediated cartilage differentiation and *in situ* deposition of ECM by BMMSCs, leading to enhanced regeneration of hyaline cartilage accompanied by reduced fibrous tissue formation, thus promoting cartilage repair (Liu H. et al., 2018). In addition, Zhang et al. used a poly (N-isopropylacrylamide-co-acrylic acid) derivative, covalently bonded to hydrolyzable degradable crosslinkers, N,O-dimethylacrylamide hydroxamide, as a carrier to support MSCs, and injected the hydrogel into the joints of OA rats. This hydrogel promoted the expression of chondrogenesis-related genes and ECM, induced chondrogenesis, and relieved cartilage defects (Zhang et al., 2020).

#### 5.2.2 Adipose stem cells (ADSC)

Adipose stem cells (ADSCs) are pluripotent cells that can be obtained from healthy donors in a minimally invasive manner and are considered important stem cells in regenerative medicine (Bhattacharjee et al., 2022; Yuan et al., 2021). Due to the complex microenvironment of the lesion site, injection of ADSCs alone may lead to their loss of function or even inactivation. To overcome these limitations, delivery systems capable of maintaining the survival and function of implanted cells are needed. These systems stimulate endogenous regeneration by promoting the interaction between transplanted cells and host tissues (Bhattacharjee et al., 2022). Wei et al. prepared an injectable, ECM-mimicking hydrogel as a cell delivery carrier, providing a favorable microenvironment for ADSC diffusion and proliferation. In a surgically induced rat model of OA, intra-articular injection of ADSC-containing hydrogels significantly reduced cartilage degradation, joint inflammation, and subchondral bone loss (Yu et al., 2021). Hyaluronic acid hydrogel microparticles (HMPs) are used to encapsulate exosomes secreted by ADSCs to prepare an injectable, sustained-release local drug delivery system. This system can prolong the retention time of the exosomes, enhance biocompatibility, promote ECM synthesis in chondrocytes, and facilitate the repair of damaged cartilage in OA (Yin Z. et al., 2023).

#### 5.2.3 Umbilical cord blood stem cells

Human cord blood-derived MSCs (hUCB-MSCs) are isolated non-invasively and have a high proliferative capacity to provide sufficient cells for therapeutic applications, (Kern et al., 2006). Experiments have also shown that hUCB-MSCs seeded on polylactic-glycolic acid copolymer scaffolds can promote cartilage regeneration in rabbit models with cartilage defects, (Lin et al., 2015). In addition, Piao et al. embedded hUCB-MSCs in hyaluronic acid hydrogel and injected them into the cartilage defect sites of osteoarthritis in elderly patients; this treatment effectively promotes cartilage regeneration and joint repair (Park et al., 2017).

### 5.3 Growth factors (GFs)

Growth factors (GFs) are effective yet sensitive therapeutic compounds that can stimulate the growth of specific tissues. Studies have found that various GFs, such as insulin-like growth factor 1 (IGF-1), transforming growth factor β (TGF-β), and TGF-β3-loaded compounds, can be used in the treatment of OA (Patil et al., 2011). However, GF therapy still faces problems such as difficulty in controlling the release kinetics profile and rapid clearance by the immune system. These issues limit its wide application (Takematsu et al., 2023). Therefore, as a material with good biocompatibility, hydrogels can overcome these drawbacks and become ideal carriers for GFs. Hydrogels not only provide a biocompatible microenvironment but also control the release rate of GFs by regulating the physicochemical properties of the hydrogels themselves, thereby achieving more effective therapeutic effects (Hiruthyaswamy et al., 2025; Salama et al., 2025).

The interpenetrating polymer network (IPN) hydrogel based on gelin-SH and polyethylene glycol diacrylate (PEGDA) forms a stable three-dimensional network through chemical and physical cross-linking. This hydrogel exhibits good cytocompatibility and effectively supports cell adhesion and proliferation (Zou et al., 2020). Insulin-like growth factor-1 (IGF-1) is a biological stimulant that promotes chondrogenic differentiation by inducing the expression of chondrogenic markers and regulating apoptosis (Aboalola and Han, 2017). The loading efficiency of IGF-1 and its protective effect in the IPN hydrogel are critical features of the hydrogel in the treatment of osteoarthritis. Studies have shown that IPN hydrogels based on gelatin-SH and PEGDA can efficiently encapsulate IGF-1 and maintain its biological activity during the release process. The structure of this hydrogel allows IGF-1 to be released *in vivo* at a controllable rate, thereby prolonging the duration of its efficacy (Kang et al., 2024). Furthermore, the IPN hydrogel has a significant protective effect on IGF-1, preventing its inactivation under environmental factors such as variations in pH and temperature. This protection ensures the maintenance of IGF-1’s biological activity during the treatment process, thereby promoting the regeneration and repair of bone tissue (León-Campos et al., 2024).

The TGF-β family plays an important regulatory role in the process of cartilage repair. Specifically, TGF-β promotes the proliferation of chondrocytes and matrix synthesis by activating the SMAD signaling pathway. This process involves the binding of TGF-β to its receptor, thereby activating downstream SMAD proteins, which then enter the cell nucleus to regulate the expression of genes related to cartilage formation. For instance, TGF-β1 and TGF-β3 play a crucial role in the proliferation and differentiation of chondrocytes, effectively promoting the synthesis of type II collagen and other matrix components and maintaining the structure and function of cartilage (Lee et al., 2024). Calcium alginate hydrogel has become an important material in the growth factor delivery system due to its superior biocompatibility and adjustable physical properties. The calcium ion cross-linking process is essential in the formation of alginate hydrogels. This process not only affects the structure and mechanical properties of the hydrogels but also has a significant impact on the protection and release of growth factors such as TGF-β. Studies have shown that calcium ions can effectively stabilize TGF-β molecules and prevent their inactivation in organisms; thereby prolonging their biological half-life and enhancing therapeutic effects (Petrushenko et al., 2025). Research has found that TGF-β can be loaded into calcium alginate hydrogel for the treatment of OA. The use of alginate hydrogels can selectively control the delivery of TGF-β to the injured site. This targeted delivery promotes the repair of damaged articular cartilage and avoids systemic side effects (Mierisch et al., 2002; Wang P. et al., 2021). TGF-β3 is an important member of the TGF-β family, which can significantly enhance the synthesis of collagen and glycosaminoglycans by chondrocytes and promote the formation of cartilage matrix (Du et al., 2023). In the treatment of OA, the hydrogel combination of hyaluronic acid (HA) and TGF-β3 has shown good application potential. HA hydrogels can continuously release TGF-β3 by controlling their crosslinked structure and network porosity (Shen et al., 2020). This continuous release can effectively maintain the biological activity of TGF-β3 and promote cell proliferation and differentiation. Researchers found that by adjusting the crosslinking degree of HA hydrogels, the release time of TGF-β3 could be significantly prolonged, thereby providing more durable growth factor support in the treatment of osteoarthritis. In addition, this hydrogel has good biocompatibility and promotes the synthesis of chondro-specific matrix and collagen, further enhancing its ability to repair defects (Fan et al., 2020). However, hydrogels have demonstrated significant advantages as growth factor (GF) carriers in the treatment of OA, including controlled release and protective activity. Despite these benefits, their application still faces limitations, such as uncontrollable release kinetics, the loss or degradation of growth factor activity in the *in vivo* microenvironment, and the difficulty in precisely regulating the spatiotemporal release sequence of different growth factors. Future breakthroughs should focus on intelligent material design, mechanical properties tailored to different scales, and optimization of clinical-grade production processes. These advances will help transition from merely providing “carrier function” to achieving true “therapeutic effectiveness.”

The above-mentioned studies indicate that injectable hydrogels are an effective drug delivery system capable of delivering various substances, such as cells, drugs, and biomolecules, for the treatment of OA (Table 1). Injectable hydrogels have advantages such as minimally invasive, local persistence, controllable combined drug delivery and promotion of tissue repair in this combined treatment of OA. They can overcome the problem of limited efficacy of single treatment methods and are an ideal platform for achieving multi-target comprehensive intervention in OA. However, co-loading systems typically provide stronger therapeutic effects compared to single drug carrier systems. By co-loading multiple active molecules (such as drugs, natural compounds, or growth factors) in hydrogels, a synergistic effect can be achieved, leveraging their respective advantages to enhance overall therapeutic efficacy (Jia et al., 2024; Quagliariello et al., 2016). Furthermore, co-loading systems can reduce the issues of mutual interference or premature release of drugs by precisely controlling the spatiotemporal sequence of different drug releases, thereby enhancing the continuity and specificity of treatment (Sattari et al., 2020). In summary, a well-designed co-loading system can optimize the bioavailability of drugs and improve therapeutic effects, especially showing significant advantages in the treatment of chronic diseases such as OA (Handali et al., 2019).

**TABLE 1:** Application of hydrogels as delivery systems in OA.

Encapsulated substances	Hydrogel composition	Specific substances	Function	References
Stem cells	Polyethylene glycol polypeptide triblock copolymer hydrogel	BMMSCs	Promote cartilage differentiation of BMMSCs and *in-situ* deposition of ECM, promotes cartilage regeneration	Liu et al. (2018b)
	Poly (n-isopropylacrylamide-co-acrylic acid) derivatives	MSCs	Induce chondrogenesis and relieve cartilage defects	Zhang et al. (2020)
	ECM simulates hydrogels	ADSCs	Reduces cartilage degradation, joint inflammation and subchondral bone loss	Yu et al. (2021)
	Hyaluronic Acid	ADSCs	Promote ECM synthesis in chondrocytes and promote cartilage repair	Yin et al. (2023b)
	Hyaluronic Acid	hUCB-MSCs	Inhibits inflammation, promotes cartilage regeneration and joint repair	Lin et al. (2015)
	Hyaluronic Acid	hUCB-MSCs	Promotes cartilage regeneration and joint repair	Park et al. (2017)
Drugs	Chitosan and Plannick-F127	Dexamethasone	Inhibits inflammation, synovitis, bone destruction and cartilage destruction	García-Couce et al. (2022)
	Alginate and Poloxam	NSAIDs	Reduces inflammation and the destruction of cartilage and bone, and promotes the formation of osteoarthritis chondrocytes	Qi et al. (2016), Chen et al. (2017b)
	Hyaluronic acid-calcium complex (HA-Ca) and sodium alginate (Alg-Na)	Icariin	Inhibit the degradation of cartilage matrix, reduce inflammation and relieve pain, and protect cartilage cells	Zhu et al. (2022b)
	Poly (ethylene glycol) dimethacrylate - gelatin methacrylate	Curcumin	Induces cartilage regeneration and promotes the repair of cartilage damage	Sun et al. (2022)
Growth factors	Gelatin -SH/PEGDA IPN hydrogel	IGF-1 and ADSC	Promote cartilage formation and ECM deposition, and enhance cartilage tissue regeneration	Cho et al. (2020)
	Hyaluronic acid (HA) gel	TGF-β3	Improve cartilage microenvironment and regeneration of cartilage defects	Lee et al. (2024)
	calcium alginate hydrogel	TGF-β	Improve the repair of articular cartilage defects	Mierisch et al. (2002)

## 6 Therapeutic mechanism of injectable hydrogels in OA

### 6.1 Anti-inflammatory response

Polylactide polyethylene glycol polylactide (PLGA-PEG-PLGA) has excellent plasticity and good biocompatibility. It can encapsulate drugs and cells and can be designed as a reservoir to control the release of therapeutic compounds. At the same time, it can solve the problem of systemic side effects of oral drugs and the short duration of efficacy of drugs injected directly into the joint (Yu et al., 2010; Zhou et al., 2023). Interleukin-36 receptor antagonists (IL-36Ra) can effectively control inflammatory responses, thereby protecting cartilage and slowing down the development of OA (Yuan et al., 2019). Yi et al. constructed a PLGA-PEG-PLGA hydrogel loaded with IL-36Ra for the treatment of OA in a mouse model. The injectable hydrogel loaded with IL-36Ra was injected into the knee joints of OA mice, and the hydrogel acted as a drug reservoir to slowly release IL-36Ra and maintain local drug concentration to effectively control inflammation. The hydrogel also adheres to the lesion site, acting as a lubricant to maintain the surface integrity of articular cartilage, reduce the degradation of cartilage matrix, and promote cartilage formation, thus effectively delaying the progression of degenerative OA changes (Yi et al., 2023). In addition, PLGA-PEG-PLGA hydrogel can also be loaded with flurbiprofen to enable continuous drug release in the joint cavity in a rat knee OA model induced by collagenase II. This approach inhibits OA inflammation by reducing the levels of pro-inflammatory cytokines (IL-1β, IL-6, and TNF-α) (Li P. et al., 2020). Furthermore, Dong developed a novel injectable hydrogel based on double crosslinking via Schiff base bonds and catechol-Fe coordination. This hydrogel stimulated the HIF-1α signaling pathway and inhibited inflammation, thus promoting cartilage differentiation. The hydrogel loaded with dexamethasone can exert anti-inflammatory effects to promote cartilage repair (Dong et al., 2022).

### 6.2 Anti-oxidative stress

Oxidative stress is an important pathological process in OA. Oxidative stress prevents chondrocytes from binding to the ECM, thus leading to chondrocyte apoptosis (Olofsson et al., 2003; Altay et al., 2015). Excessive ROS can serve as an important intracellular signaling molecule. It enhances inflammation in joints, promotes chondrocyte death, and leads to joint injury (Bordy et al., 2018; Mateen et al., 2016). Hydrogel scaffolds have been reported to effectively clear ROS (Cheng et al., 2017). GHC hydrogel (composed of gelatin, hyaluronic acid, and chondroitin sulfate) possesses multifunctional properties, including tissue adhesion ability, anti-ROS function, and the ability to promote cartilage formation, making it an ideal cartilage repair material (Tong et al., 2024). Polycitrate-based materials, specifically PCCGA hydrogels as an emerging biomaterial, have shown great potential in the treatment of osteoarthritis. The self-polymerization property of PCCGA hydrogel enables it to form a stable three-dimensional network structure *in vivo*, providing excellent biocompatibility and adjustable physicochemical properties. Therefore, it has received extensive attention in cartilage repair and regeneration (Xia et al., 2025). PCCGA hydrogel also inhibits the expression of matrix metalloproteinase-13 (MMP-13) by regulating the redox state inside and outside cells. MMP-13 is a key enzyme closely related to cartilage degradation. Excessive MMP-13 can lead to the degradation of cartilage matrix and accelerate the progression of osteoarthritis (Wang et al., 2024). A study using PCCGA hydrogel to treat OA mice found that the hydrogel could significantly reduce the expression of MMP-13, thereby protecting chondrocytes from damage by inflammatory mediators and promoting the recovery of cell metabolic function (He et al., 2024). Epigallocatechin-hyaluronic acid (EGCG-HA) hydrogel, as a new type of long-acting injectable carrier, has received extensive attention in the treatment of OA in recent years. This hydrogel not only has excellent biocompatibility but also effectively improves the microenvironment within the OA joint through its powerful antioxidant properties (Jin et al., 2020). Li et al. found that injecting EGCG-HA hydrogel into the joints of OA rats could significantly induce synovial macrophages to polarization into the M2 phenotype, reduce the expression of pro-inflammatory cytokines (such as IL-1β, MMP-13, and TNF-α), and thereby promote the formation of cartilage matrix and repair damaged cartilage tissue in the OA model (Li et al., 2022). In addition to the above-mentioned hydrogels, hydrogen-releasing hydrogels (Zhang et al., 2023), selenium nanoparticle-loaded hydrogels (Hu et al., 2023), and siMMP13-loaded liposome hydrogels (Ji Z. et al., 2024) can also eliminate ROS and reduce the expression of related inflammatory cytokines, thereby promoting cartilage repair.

### 6.3 Promoting cartilage regeneration

**FIGURE 5:**
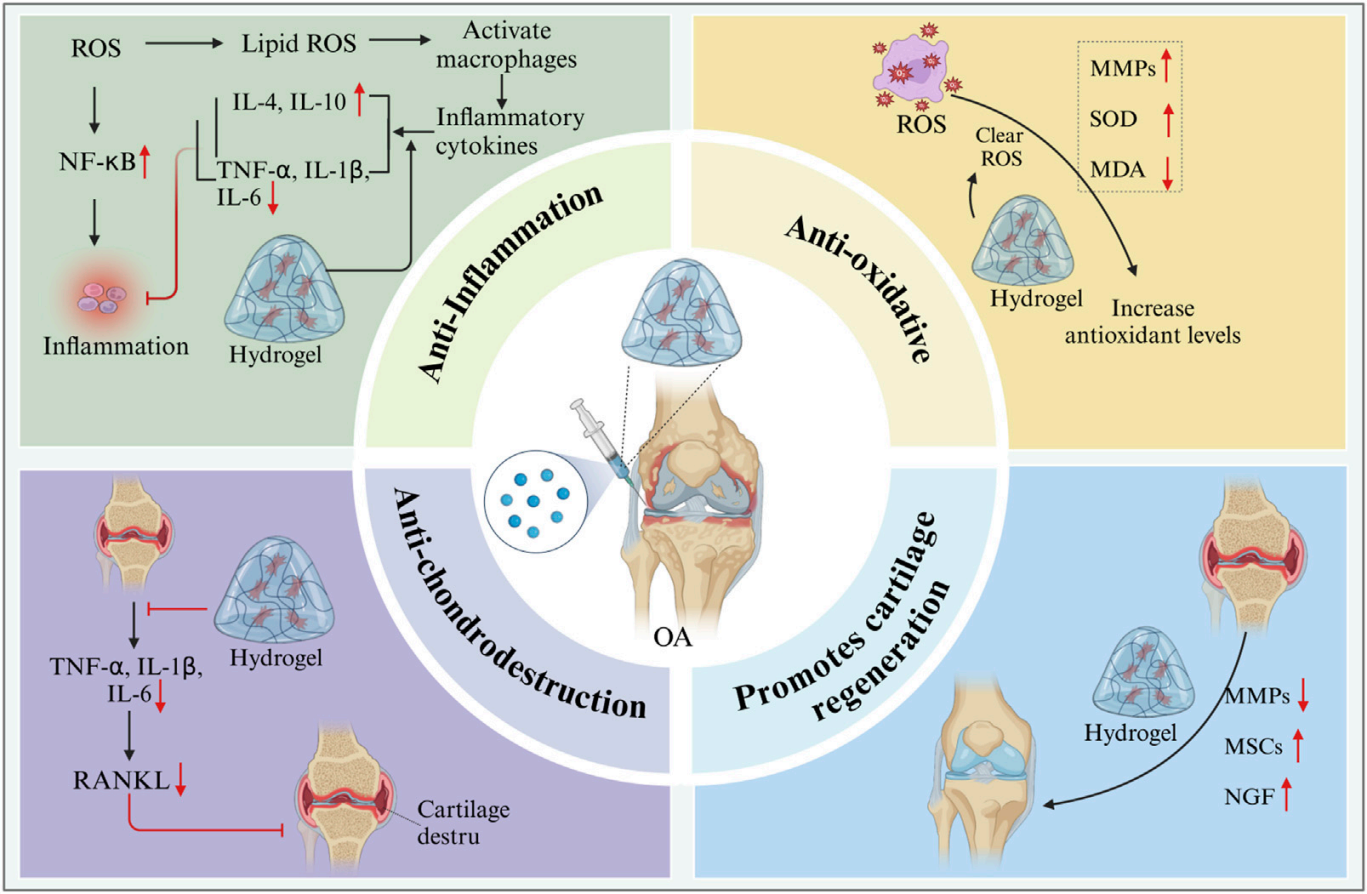
Schematic diagram of preparation of hydrogel drug delivery system for OA by intra-articular injection. The image is drawn using the BioRender software.

Cartilage destruction is caused by matrix metalloproteinases produced by chondrocytes, synovial fibroblasts, and synovial macrophages, as well as disintegrating proteins and metalloproteinases with platelet protein motifs (Araki et al., 2016). Currently, treatment options for cartilage repair are very limited, and conventional drug therapy cannot restore damaged cartilage. Given the various properties of injectable hydrogels, their development for cartilage repair may be an effective strategy (Levinson et al., 2019). Platelet-derived growth factor-BB (PDGF-BB) has been found to significantly reduce the apoptosis rate of chondrocytes and promote the migration of cells to the injury site, which is crucial for cartilage self-repair (Zhu et al., 2021). Li et al. constructed a bioactive injectable porous hydrogel microsphere with sustained paracrine signaling activity through freeze-drying microfluidic technology by combining PDGF-BB and exogenous MSCs. Injecting this hydrogel into the joint cavity of OA rats can effectively promote the interactions between cells and ECM, as well as between cells, and enhances paracrine signaling, thereby promoting the regeneration of articular cartilage (Li X. et al., 2023). Bone morphogenetic protein-7 (BMP-7) is an important growth factor that has been widely studied for cartilage formation and regeneration. Kalairaj MS et al. discovered that incorporating BMP-7 into a novel polymer hydrogel enhances its biocompatibility and drug release characteristics, effectively promotes the differentiation of chondrocytes, and significantly improves the repair efficacy for cartilage defects in in vivo experiments (Kalairaj et al., 2024). This combined strategy offers a new approach for cartilage regeneration, especially in clinical applications, where it can enhance the success rate and efficiency of cartilage repair by precisely regulating the release and action of growth factors (Figure 5).

Additionally, researchers have developed a hydrogel (T-GAG) cross-linked from hyaluronic acid (HA) and aggrecan, with adjustable mechanical properties that mimic the viscoelastic characteristics of cartilage. This study indicates that the aggregation coefficient (compressive modulus) of T-GAG hydrogel can be controlled by adjusting the concentrations of HA and aggrecan, and at certain concentrations, its aggregation coefficient can reach or exceed the literature-reported values for articular cartilage. Furthermore, T-GAG hydrogel exhibited a characteristic tension relaxation response typical of biphasic materials (such as cartilage) in closed compressive tests, demonstrating its mechanical properties similar to those of articular cartilage (Mintz and Cooper, 2014; Han et al., 2010). Although the aforementioned research provides preliminary data on hydrogels simulating cartilage ECM, there is still a lack of studies comparing *in vitro* test results with actual joint loading conditions. To better assess the performance of hydrogels in the joint environment, future research should consider combining *in vitro* tests with *in vivo* models for more accurate evaluation and optimization. For example, animal models or biomechanical simulations could be used to simulate the effects of joint movement on hydrogels, thus enabling a more accurate assessment of their performance in practical applications (Qiu et al., 2025; Almeida et al., 2016).

## 7 Challenges and prospects of injectable hydrogels in the treatment of OA

### 7.1 Current research challenges

#### 7.1.1 Regulation of stability and degradability of hydrogels

In the treatment of OA, the regulation of stability and degradability of hydrogels is of vital importance, but it also faces many challenges. On the one hand, hydrogels need to maintain sufficient stability in the body to ensure that they can continuously exert therapeutic effects. During the drug delivery process, hydrogels need to maintain structural integrity to ensure that drugs are released at the expected rate. If the stability of the hydrogel is insufficient, it may disintegrate before the drug is fully released, resulting in rapid drug leakage, thus failing to achieve long-term treatment. For some hydrogels used to repair cartilage defects, a stable structure can provide continuous support for the growth and proliferation of chondrocytes and promote the regeneration of cartilage tissue. If the hydrogel degrades prematurely, chondrocytes will lose their suitable growth environment, affecting the cartilage repair effect. Conversely, after the treatment is completed, the hydrogel should be able to degrade and be eliminated from the body in a timely manner to avoid remaining in the body and causing adverse reactions. However, at present, it is often difficult to balance the stability and degradability of the hydrogel simultaneously.

In addition, the matching of degradation rate and union rate is also a crucial factor in the design of hydrogels (Erickson et al., 2020). The ideal hydrogel should be able to match the process of cartilage repair, with its degradation rate coordinated with the healing speed of cartilage tissue. If the degradation of the hydrogel is too fast, it may lead to the premature loss of its supportive function, affecting cartilage regeneration. Conversely, if the degradation is too slow, it may result in hydrogel residue, triggering immune responses or affecting normal joint function (Lalitha Sridhar et al., 2017). Therefore, optimizing the degradation rate of hydrogels to ensure they provide sufficient support for chondrocytes during the repair process and can degrade in a timely manner after cartilage healing is an important consideration in the design of hydrogels.

To solve this difficult problem, researchers have attempted to regulate the stability and degradability of hydrogels through various methods. In terms of material selection, optimizing the structure and composition of polymer materials is key. For instance, chemical modification of natural polymers can alter the structure and properties of their molecular chains. Quaternization of chitosan can enhance its stability; moreover, by controlling the degree of modification, its degradation rate can be regulated. In terms of the preparation process, precisely controlling the type and concentration of crosslinking agents can effectively regulate the crosslinking density of hydrogels, thereby influencing their stability and degradability. Increasing the concentration of crosslinking agents usually enhances the stability of hydrogels, but it reduces their degradation rate. Studying the influence of different crosslinking agents on the performance of hydrogels and screening the most suitable crosslinking system for the treatment of osteoarthritis are also current research focuses. Additionally, developing hydrogels with environmental responsiveness is an effective strategy. By taking advantage of changes in environmental factors such as temperature, pH value, and enzymes, the stability and degradability of hydrogels can be intelligently regulated. At the site of joint inflammation, the pH value is usually low. Designing pH-responsive hydrogels enables these materials to accelerate degradation in an acidic environment, ensuring stability under normal physiological conditions while also degrading promptly after application.

#### 7.1.2 Large-scale preparation and quality control

For injectable hydrogels to move from laboratory research to clinical application, large-scale preparation and quality control are the major challenges that must be overcome. Among these challenges, ensuring process repeatability during large-scale preparation is critical. During laboratory preparation, hydrogels are usually prepared under conditions of small volume and precise control. However, during large-scale production, it is difficult to fully reproduce these conditions. Because factors such as the temperature of the reaction system, the stirring speed, and the uniformity of raw material mixing are difficult to control precisely in large-scale production, variations in mechanical properties and functional performance arise in hydrogels prepared from different batches. These variations may affect the therapeutic effect and safety of hydrogels, limiting their clinical application. During large-scale preparation of alginate hydrogel, ensuring the exact same mixing ratio of sodium alginate and crosslinking agent in each batch is challenging, which leads to variability in degree of crosslinking and mechanical properties of the hydrogel.

The uniformity of product quality is also an important issue faced in large-scale production. The uniformity of quality of hydrogels includes the uniformity of physical properties (such as mechanical and swelling properties), chemical composition, and drug loading amount. Hydrogels with non-uniform physical properties may experience inconsistent injection resistance during the injection process, which can affect clinical operations. Moreover, variations in chemical composition can lead to differences in biocompatibility and degradability of hydrogels, thereby increasing the risk to patients. Uneven drug loading will not only affect the drug release behavior but also the therapeutic effect. In the preparation of hydrogels loaded with drugs, if the drugs are not evenly distributed, it may lead to excessively high drug concentrations in some areas, causing toxicity and adverse side effects. Meanwhile, in other areas, the drug concentrations may be too low to achieve therapeutic effects.

At present, the application of industrial production technology in the preparation of injectable hydrogels is not yet mature. The traditional methods for preparing hydrogels are often inefficient and difficult to meet the demands of large-scale production. However, some advanced preparation technologies, such as microfluidic and 3D printing technologies, show good application prospects in the laboratory. Nevertheless, they still face many problems during industrial scale-up. When preparing hydrogels using microfluidic technology, how to achieve large-scale fabrication and integration of microchannels as well as how to improve production efficiency and reduce costs are urgent problems to be solved. 3D printing technology has advantages in preparing complex-structured hydrogels; however, it is slow in printing speed, results in significant material waste, and may introduce impurities during the printing process, which affects the quality of the hydrogel.

To achieve large-scale preparation and quality control of injectable hydrogels, it is necessary to enhance the research and optimization of the preparation process. It is necessary to develop automated and intelligent preparation equipment, precisely control reaction conditions, and enhance the repeatability and stability of the preparation process. Establish strict quality control standards and testing methods to conduct comprehensive tests on the physical properties, chemical composition, and drug loading capacities of hydrogels, ensuring the uniformity of product quality. Strengthen cooperation with the industrial sector, introduce advanced industrial production technologies into the field of hydrogel preparation, and promote the industrialization process of injectable hydrogels.

#### 7.1.3 Clinical translational disorders

Injectable hydrogels face numerous challenges in safety evaluation and regulatory approval as they transition from laboratory research to clinical application. Although injectable hydrogels have demonstrated good biocompatibility in both *in vitro* and animal experiments, uncertainty remains about how well these safety data can be extrapolated from animal models to humans. The physiological environment and immune system within humans are more complex, and the long-term safety of hydrogels *in vivo* still requires verification. Moreover, the metabolic pathways and potential toxicity of hydrogel degradation products in the human body remain unclear. These degradation products may have toxic, immunogenic, or other harmful effects on human health. For example, some hydrogels may produce small molecular weight compounds during degradation. Whether these compounds cause damage to vital organs such as the liver and kidneys warrants further in-depth research.

Clinical trial design also faces many challenges. Determining the appropriate endpoints of clinical trials is one of the key issues. At present, the clinical trial endpoints of osteoarthritis mainly include the extent of pain relief, the improvement in joint function, and changes in imaging biomarkers. However, these indicators are often influenced by multiple factors and vary significantly among different patients; this leads to a certain degree of subjectivity and uncertainty in the evaluation of clinical trial results. The extent of pain relief is primarily assessed based on the patient’s subjective reporting. Different patients have different tolerance levels and ways of describing pain, which may affect the accuracy of the assessment of pain relief. Selecting objective, accurate, and quantifiable clinical trial endpoints is an urgent problem to be solved. This would allow a more scientific evaluation of the therapeutic effect of injectable hydrogels. Moreover, clinical trials need to take into account factors such as sample size, control group setting, and trial duration. As osteoarthritis is a chronic disease with a long course, it requires long-term clinical trial observation. This not only increases the cost and difficulty of the trial but may also lead to a decline in patient compliance, affecting the reliability of the trial results. Moreover, reasonable setting of the control group is also an important part of clinical trial design. The selection of appropriate control treatment methods, such as placebo controls and existing standard treatment controls, requires a comprehensive consideration of multiple factors, including ethics and clinical practice.

In terms of regulatory approval, as a new type of biomaterial, the relevant regulations and standards for injectable hydrogels are still incomplete at present. The approval requirements for biomaterials vary among different countries and regions, which poses difficulties for the global promotion of injectable hydrogels. The regulatory approval process is usually rather complicated and requires a large amount of experimental data and numerous materials. These include material preparation processes, quality control, safety evaluation, and clinical trial results. Moreover, for complex biomaterials like injectable hydrogels, meeting the requirements of regulatory approval and accelerating the approval process are key to clinical translation. The approval standards in some countries focus on safety, while others pay more attention to effectiveness. Enterprises need to formulate corresponding regulatory application strategies in accordance with the regulatory requirements of different countries and regions, which increases the costs and difficulties of research, development, and promotion.

### 7.2 Future outlook

#### 7.2.1 Material design and performance optimization direction

In the future, injectable hydrogels are expected to make significant breakthroughs in material design, and the development of new intelligent response materials will become a research hotspot. With the in-depth understanding of the pathogenesis of osteoarthritis and the microenvironment of joints, it has become possible to develop hydrogels that can respond to various stimuli. In addition to the common responses to temperature and pH values, hydrogels can also respond to specific molecules in the joint microenvironment such as inflammatory factors and reactive oxygen species. When the concentration of inflammatory factors in the joint cavity increases, the hydrogel can rapidly release anti-inflammatory drugs, which precisely inhibit the inflammatory response. This multi-responsive hydrogel can intelligently adjust the drug release profile according to the real-time changes of the joint microenvironment, improving the targeting and effectiveness of treatment.

Optimizing the mechanical properties and biological activities of materials is also an important development direction in the future. Regarding mechanical properties, it is necessary to develop hydrogels with adaptive mechanical properties, enabling them to automatically adjust their mechanical strength under different joint movement states to better meet the mechanical requirements of joints. Hydrogels can enhance their mechanical strength to provide sufficient support when joints bear weight. When the joints are in motion, hydrogels can maintain good flexibility and not affect the normal movement of the joints. By introducing materials with special mechanical properties, such as shape memory polymers and self-healing materials, the mechanical properties of hydrogels can be adaptively regulated. ​

In terms of biological activity, it is important to further enhance the affinity of hydrogels for cells and their ability to promote cell functions. Introducing more bioactive molecules, such as growth factors, cell adhesion peptides, and signaling pathway activators, into the hydrogel can construct a system with multifunctional bioactivity. These bioactive molecules can interact with receptors on the cell surface, activate intracellular signaling pathways, promote the proliferation and differentiation of chondrocytes, and the synthesis of extracellular matrix, thereby accelerating cartilage repair.

At the same time, the optimization of biodegradation kinetics will become a key factor in material design. The degradation rate of hydrogels should be reasonably designed to ensure that they maintain sufficient mechanical strength during the treatment process and degrade at the appropriate time, thereby promoting sustained therapeutic effects and tissue repair (Cota Quintero et al., 2025). Regarding bioactivity, the affinity of hydrogels for cells and their ability to enhance cell function should be further improved. To this end, more functional biomolecules, such as growth factors, cell adhesion peptides, and signaling pathway activators, should be introduced into hydrogels to construct a multifunctional bioactive hydrogel system. These molecules can interact with receptors on the cell surface and activate intracellular signaling pathways. They also promote the proliferation, differentiation, and extracellular matrix synthesis of chondrocytes, thereby accelerating cartilage repair.

By integrating artificial intelligence and machine learning technologies, the material properties of injectable hydrogels can be designed and optimized more efficiently. Specifically, by establishing a mathematical model between the structure and properties of hydrogels, and using machine learning algorithms to analyze and predict large datasets of experimental data, hydrogel materials and formulations with ideal properties can be quickly screened out. Artificial intelligence can also assist in designing the microstructure of hydrogels, endowing them with better drug loading and release performance and enhanced cell biocompatibility. By simulating the behavior of hydrogels with different structures *in vivo*, the hydrogels’ therapeutic effects can be predicted, providing a scientific basis for the optimal design of hydrogels. ​

#### 7.2.2 Trend of interdisciplinary integration

The integration of multiple disciplines such as biomedicine, materials science, nanotechnology, and gene therapy will bring unprecedented opportunities for the application of injectable hydrogels in the treatment of osteoarthritis. In the field of biomedicine, in-depth research on the pathogenesis of osteoarthritis can provide more precise targets and therapeutic strategies for the design and application of injectable hydrogels. Through research on cytokines, signaling pathways, gene expression, and other aspects in the microenvironment of osteoarthritis joints, new therapeutic targets are discovered, and injectable hydrogel drug delivery systems capable of targeting them are developed. Understanding the aberrant expression of specific genes in osteoarthritis allows the design of hydrogels capable of carrying related gene therapy drugs to regulate disease-related genes and address the underlying causes of OA.

The development of materials science has provided novel materials and preparation techniques for injectable hydrogels. The synthesis and modification of new polymer materials can endow hydrogels with enhanced mechanical strength, biocompatibility, and degradation profiles. Researchers develop polymer materials with higher biocompatibility, mechanical properties, and degradation performance to lay the foundation for the application of hydrogels. Advanced preparation techniques, such as electrospinning, microfluidics, and 3D printing, can precisely control the microstructure and macroscopic shape of hydrogels, enabling personalized customization. Using 3D printing technology, injectable hydrogel scaffolds that match the specific morphology and cartilage defects of the patient’s joints can be printed, thereby enhancing the therapeutic effect.

The combination of nanotechnology and injectable hydrogels can further enhance the performance and functionality of hydrogels. Nanomaterials possess unique physical and chemical properties, such as high specific surface area, small size effect, and quantum size effect. Introducing nanomaterials such as nanoparticles, nanofibers, and nanotubes into hydrogels can enhance their mechanical properties, drug loading capacity, and targeting capabilities. The incorporation of nano hydroxyapatite into hydrogels can enhance their biological activity and mechanical strength, as well as promote the adhesion and proliferation of chondrocytes. Targeted nanocarriers developed using nanotechnology, when combined with injectable hydrogels, can achieve precise drug delivery, improve therapeutic effects, and reduce drug side effects.

The combination of nanotechnology and injectable hydrogels can further enhance the performance and functionality of hydrogels. Nanomaterials possess unique physical and chemical properties, such as high specific surface area, nanoscale size effects, and quantum size effects. Introducing nanomaterials—such as nanoparticles, nanofibers, and nanotubes-into hydrogels can improve their mechanical strength, drug loading capacity, and targeting capabilities. For example, the incorporation of nano-hydroxyapatite into hydrogels can enhance their biological activity and mechanical strength, as well as promote the adhesion and proliferation of chondrocytes. Moreover, targeted nanocarriers developed using nanotechnology combined with injectable hydrogels enable precise drug delivery, improve therapeutic effects, and reduce drug side effects.

## 8 Conclusion

Osteoarthritis (OA) is a common degenerative joint disease that can involve various pathological changes such as cartilage degradation, synovitis, and subchondral bone remodeling. The current treatment options for OA aim to alleviate pain and disease activity, and to prevent inflammation and the progression of destructive processes. However, due to the complex pathological processes and local microenvironment of OA, as well as the limited variety of treatment methods and the low efficiency of traditional administration routes, achieving satisfactory therapeutic effects remains challenging. In this context, the emergence of advanced injectable hydrogels offers a promising approach to overcoming these limitations. Injectable hydrogels are a unique type of hydrophilic polymer characterized by a cross-linked three-dimensional network structure. They feature excellent mass transport capacity, biocompatibility, biodegradability, and adjustable mechanical properties. These unique properties make them effective functional matrices for drug delivery. Injectable hydrogels enable the controlled release of drugs as well as tissue repair factors, providing a promising therapeutic platform for the treatment of OA.
